# The role of microRNAs in non-invasive diagnosis of bladder cancer: a systematic review

**DOI:** 10.31744/einstein_journal/2024RW0611

**Published:** 2024-11-06

**Authors:** Pedro Ivo de Sousa, Vicktor Bruno Pereira Pinto, Elaine dos Santos Piancó, Malene Lima Gomes, Sally Cristina Moutinho Monteiro, Flávia Castello Branco Vidal, Maria do Desterro Soares Brandão Nascimento, Jaqueline Diniz Pinho, José de Ribamar Rodrigues Calixto, Marcelo Souza de Andrade

**Affiliations:** 1 Universidade Federal do Maranhão São Luís MA Brazil Adult Health Postgraduate Program, Universidade Federal do Maranhão, São Luís, MA, Brazil.; 2 Universidade Estadual do Maranhão Zé Doca Center for Higher Studies Zé Doca MA Brazil Zé Doca Center for Higher Studies, Universidade Estadual do Maranhão, Zé Doca, MA, Brazil.; 3 Universidade Federal do Maranhão Hospital Universitário São Luís Brazil Hospital Universitário, Universidade Federal do Maranhão, São Luís, Brazil.

**Keywords:** Urinary bladder neoplasms, Niomarkers, Early detection of cancer

## Abstract

**Objective:**

MicroRNAs are small non-coding RNAs that are abundantly expressed in various biofluids, making them promising candidates for cancer biomarkers. This review aims to present current evidence on the use of miRNA as biomarkers for the non-invasive diagnosis of bladder cancer.

**Methods:**

A systematic literature review, using the Medline database, was performed in July 2022 according to the Preferred Reporting Items for Systematic Reviews and Meta-Analyses guidelines. All articles were required to satisfy the risk-of-bias assessment using the Joanna Briggs Institute Critical Assessment Tools. Data were collected based on miRNA expression, sample type, expression profiles, and accuracy.

**Results:**

The initial search retrieved 437 studies, 21 of which were included in the final analysis. Most studies on miRNA expression in human fluids used urine samples for analysis.

**Conclusion:**

There is a trend to cluster the expressed miRNAs to build diagnostic panels or use them in association with other diagnostic methods to achieve reasonable accuracy.

**Prospero database registration:** (https://www.crd.york.ac.uk/prospero/) under ID CRD42022351686.

## INTRODUCTION

Bladder cancer (BCa) is the ninth most common cause of tumor-related death, affecting approximately 3.4 million people worldwide.^(1)^ Early detection of BCa is essential for improving patient prognosis and survival rates.^(2)^ However, the methods used in clinical practice are often unsatisfactory because they are inaccurate or highly invasive.^(3)^

Although limited in number, there are a few standardized non-invasive markers for diagnosing BCa that can replace invasive procedures such as cystoscopy. Voided urine cytology (VUC) is the primary method used for detecting BCa using urine samples. Voided urine cytology has high specificity but low sensitivity, especially in low-grade tumors.^(4)^ Consequently, alternative biomarkers have been investigated to overcome this disadvantage.^(5)^

MicroRNAs (miRNAs) are small non-coding RNAs that are conserved throughout evolution and regulate gene expression.^(6)^ Depending on the context and cell type in which they are expressed, the same miRNAs may exhibit oncogenic or tumor-suppressive activities.^(7)^ Circulating miRNAs are abundant in various biofluids including blood and urine, making them promising candidates as cancer biomarkers.^(8)^

miRNA profiles have been associated with several types of tumors, including BCa.^(4)^

## OBJECTIVE

This review aims to present the main current evidence on the use of miRNAs as biomarkers for the non-invasive diagnosis of bladder cancer.

## METHODS

### Protocol and registration

This systematic review was conducted in accordance with PRISMA guidelines.^(9)^

### Data source and search strategy

Two independent reviewers searched the MEDLINE database using the same search strategy, including papers from the last five years, with no restriction on the language of publication. The database was searched for articles published before July 2022. In the literature search, each of the terms for exposure was combined with the outcome term, using Boolean terms, as follows: (((urothelial cancer) OR (bladder cancer)) AND ((microRNA) OR (miRNA)) AND ((screening) OR (diagnostic))).

### Eligibility criteria

Observational studies that compared miRNA expression in patients with BCa or urothelial upper tract urinary cancer and in controls without these cancers were included in this study. In addition, these studies were required to assess the use of the identified biomarkers as a potential tool for non-invasive diagnosis of the disease.

For study eligibility, the patient population, intervention, comparator, outcome, and study design (PICOS) approach was used. A study was considered relevant if it met the following criteria: patients diagnosed with BCa or urothelial cancer (P); miRNA was investigated as a potential biomarker for the non-invasive diagnosis of BCa or for improving other non-invasive diagnostic tools for neoplasia (I); controls were included for comparison (C); and the effectiveness of the biomarker (O) was assessed; and review articles, opinion articles, editorials, commentaries, case reports, and studies conducted exclusively with animals or cell lines in vitro and meeting abstracts were excluded from the review (S).

### Selection of studies

Titles and abstracts were perused to exclude irrelevant studies. The full texts of the remaining studies were retrieved, and those eligible for this systematic review were identified.

The studies were selected independently by two reviewers considering the eligibility criteria. Any disagreements were resolved by a third reviewer.

### Data extraction

Two reviewers extracted data from the included studies using a standardized protocol (accessed on the PROSPERO website). For each study, the following information was extracted: authorship and publication year, place of study (city, state, and/or country), data collection period, study design, setting (hospital or population), sample size, miRNA source (blood, urine, or tissue), and the main results of the study. Microsoft Office Excel software (version 17.0) was used to facilitate tabulation and better visualization of the study data.

### Assessment of risk of bias

The methodological quality (risk of bias) of the studies was assessed by two independent reviewers using the Joanna Briggs Institute (JBI) Critical Assessment Tool. This tool was chosen because it presents specific checklists for several study types, including case controls, to assess the trustworthiness, relevance, and results of published papers.^(10)^

## RESULTS

### Quality of studies and measure of evidence

**Figure 1:**
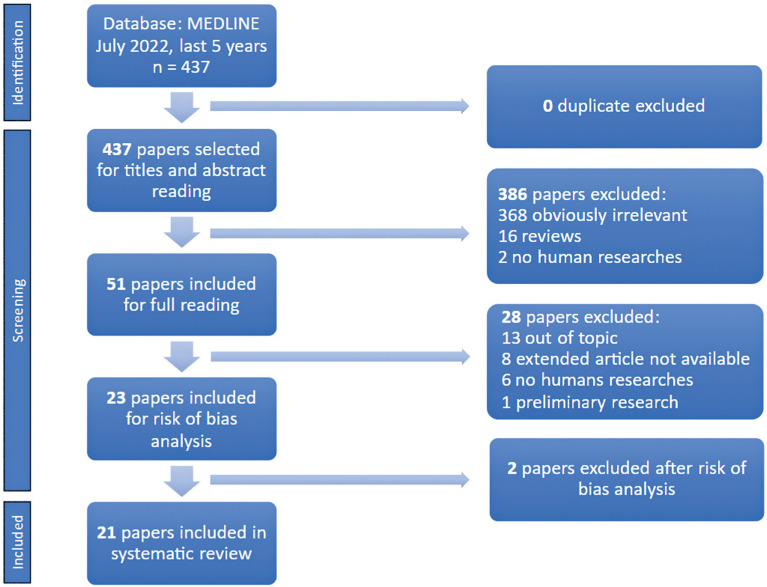
Selection of research articles for the systematic review

A total of 437 papers were retrieved by searching the MEDLINE database using the key search words (July 2022). Of these, 21 were selected for this systematic review (Figure 1). After reading the titles and abstracts, 386 articles were excluded because they did not meet the inclusion criteria. Subsequently, 28 papers were excluded after a full-text review. During the risk of bias analysis of the remaining 23 papers, 2 were excluded due to a high risk of bias. All remaining articles satisfied the risk-of-bias assessment requirements (Table 1). Of the final selection, 10 studies investigated miRNAs in urine samples, 9 investigated biomarkers in blood, and 2 in both fluids (Table 2).

**Table 1:** Quality assessment of included articles using the JBI checklist for case control studies

Studies	1	2	3	4	5	6	7	8	9	10
Wang et al, 2020^(2)^	Y	Y	Y	Y	Y	Y	Y	Y	N/A	Y
Erdmann et al, 2020^(4)^	Y	Y	Y	Y	Y	UN	UN	Y	N/A	Y
Singh et al, 2022^(6)^	Y	Y	Y	Y	Y	UN	UN	Y	N/A	Y
Lin et al, 2021^(8)^	Y	Y	Y	Y	Y	Y	N	Y	N/A	Y
Li et al, 2021^(11)^	Y	Y	Y	Y	Y	Y	Y	Y	N/A	Y
Lin et al, 2021^(14)^	Y	Y	Y	Y	Y	Y	Y	Y	N/A	Y
Tölle et al, 2019^(19)^	Y	Y	Y	Y	Y	Y	Y	Y	N/A	Y
Chen et al, 2019^(20)^	Y	Y	Y	Y	Y	Y	UN	Y	N/A	Y
Geva et al, 2017^(21)^	Y	Y	Y	Y	Y	UN	UN	Y	N/A	Y
Li et al, 2022^(22)^	Y	Y	Y	Y	Y	Y	UN	Y	N/A	Y
Juracek et al, 2018^(23)^	Y	Y	Y	Y	Y	Y	Y	Y	N/A	Y
Piao et al, 2019^(24)^	Y	Y	Y	Y	Y	Y	Y	Y	N/A	Y
Hofbauer et al, 2018^(25)^	Y	Y	Y	Y	Y	UN	UN	Y	N/A	Y
Usuba et al, 2019^(26)^	Y	Y	Y	Y	Y	Y	Y	Y	N/A	Y
Chen et al, 2019^(28)^	Y	Y	Y	Y	Y	Y	Y	Y	N/A	Y
Yang et al, 2021^(29)^	Y	Y	Y	Y	Y	Y	Y	Y	N/A	Y
El-Shal et al, 2021^(30)^	Y	Y	Y	Y	Y	Y	Y	Y	N/A	Y
Jiang et al, 2020^(32)^	Y	Y	Y	Y	Y	N	N	Y	N/A	Y
Ghorbanmehr et al, 2019^(33)^	Y	Y	Y	Y	Y	UN	UN	Y	N/A	Y
Wang et al, 2019^(34)^	Y	Y	Y	Y	Y	Y	Y	Y	N/A	Y
Wang et al, 2019^(35)^	Y	Y	Y	Y	Y	Y	Y	Y	N/A	Y

Y: yes; N: no; UN: unclear; N/A: not applicable.

**Table 2:** Characteristics of studies involving microRNAs in non-invasive diagnosis of bladder cancer

Author	Year	Patients	Control	Sample type	miRNA	Expression	AUC	Sensitivity %	Specificity %
Wang et al^(2)^	2020	20 (4+15+1)	49 (4+37+8)	Blood	miR-130a-3p	Up	0.84		
					miR-130b-3p	Up	0.76		
					miR-301a-3p	Up	0.89		
					miR-301b-3p	NA	0.70		
Erdmann et al^(4)^	2020	104	46	Urine	miR-96	Up	0.60	29.8	91.3
					miR-126	Up	0.66	88.5	21.7
					miR-183	Up	0.72	81.7	60.9
					miR-125b	Down	0.71	88.5	54.3
					miR-145	Down	0.68	50	84.8
					miR-221	Down	0.77	77.9	67.4
Singh et al^(6)^	2022	25	35 (25+10)	Blood	miR-9	Up	0.81	84	68
					miR-34a	Down	0.70	68	72
					miR-203	Up	0.72	72	72
				Urine	miR-9	Up	0.89	100	73.3
					miR-34a	NA			
					miR-203	Up	0.92	93.3	80
Lin et al^(8)^	2021	80	100	Urine	let-7b-5p	Up			
					miR-149-5p	Up			
					miR-146a-5p	Up			
					miR-423-5p	Up			
Li et al^(11)^	2021	111 (11+50+50)	111 (11+50+50)	Urine	miR-1274a	Down	0.71		
					miR-30a-5p	Down	0.64		
					miR-19a-5p	Up	0.61		
				Blood	miR-155-5p	Down	0.65		
					miR-19b-1-5p	Down	0.66		
					miR-636	Down	0.61		
Lin et al^(14)^	2021	63 (12+51)	55 (4+51)	Urine	miR-93-5p	Up	0.83	74.1	90.2
					miR-516a-5p	Up	0.79	72.9	89.9
Tölle et al^(19)^	2019	46 (10+36)	10	Blood	miR-15b-5p	Up	0.99	97.2	100
					miR-590-5p	Up	0.77	69.4	90
Chen et al^(20)^	2019	122	110	Blood	miR-101	Down	0.88	82	80.9
Geva et al^(21)^	2017	20	14	Urine	miR-210	Up	0.89	93.3	76.2
Li et al^(22)^	2022	137 (25+30+82)	127 (15+30+82)	Blood	miR-148b-3p	Up	0.83		
					miR-106a-5p	Down	0.63		
					miR-145-5p	Down	0.67		
					miR-132-3p	Down	0.78		
					miR-7-5p	Down	0.77		
Juracek et al^(23)^	2018	205	99	Urine	miR-31-5p	Up	0.78	74	73
					miR-93-5p	Up	0.80	74	72
					miR-191-5p	Up	0.76	73	68
Piao et al^(24)^	2019	326	217 (174+43)	Urine	[miR-6124, miR-4511]	[Up, Down]	0.81	78.5	70.9
Hofbauer et al^(25)^	2018	115	87	Urine	[let-7c, miR-148a, miR-204, miR-135a, miR-135b, miR-345]	[Down, Down, Down, Up, Up, Up]	0.88		
Usuba et al^(26)^	2019	392 (196+196)	580 (290+290)	Blood	miR-6087	Down	0.89	93	77
					[miR-6087, miR-1343-5p]	[Down, Down]	0.90	97	74
					[miR-6087, miR-6724-5p,miR-3960, miR-1343-5p, miR-1185-1-3p, miR-6831-5p, miR-4695-5p]	[Down, Up, Down, Down, Up, Up, NA]	0.97	95	87
Chen et al^(28)^	2019	20 (4+15+1)	49 (4+37+8)	Blood	miR-150-5p	Down			
					[miR-155-5p, miR-150-5p]		0.75		
					[miR-378a-3p, miR-150-5p]		0.71		
					[miR-636, miR-150-5p]		0.70		
					[miR-150-5p, miR-210-3p]		0.68		
					[miR-19b-1-5p, miR-378a-3p]		0.67		
					[[miR-155-5p, miR-150-5p]-[miR-378a-3p, miR-150-5p]-[miR-636, miR-150-5p]-[miR-19b-1-5p, miR-378a-3p]		0.88	80	83.7
Yang et al^(29)^	2021	208 (88+120)	36	Blood	miR-10a-5p	Up	0.78	75	64.2
El-Shal et al^(30)^	2021	51	45 (21+24)	Urine	miR-96-5p	Up	0.85	80.4	91.8
					miR-183-5p	Up	0.83	78.4	81.6
					[miR-96-5p, miR-183-5p]		0.88	88.2	87.8
Jiang et al^(32)^	2020	118	120	Urine	miRNA-192	Down	0.79	76.7	78
					[miRNA-192, BUS]		0.95	93.2	76.7
Ghorbanmehr et al^(33)^	2019	45	42	Urine	miR-21-5p	Up	0.76	84	59
					miR-141-3p	Up	0.74	71	71
					miR-205-5p	Up	0.73	82	62
Wang et al^(34)^	2019	50	40	Blood	miR-492	Up	0.86		
Wang et al^(35)^	2019	55	45	Blood	miRNA-373	Up	0.84		

AUC: area under the curve; NA: not altered; miRNA: microRNAs.

Most studies investigating diagnostic tools used the area under the receiver operating characteristic curve (AUC), which is the most commonly used performance measure to indicate the discriminative ability of a prediction mode. An AUC value >0.6 could be a potential marker and >0.7 is strong enough to differentiate between two groups.^(11,12)^ The measures of sensitivity and specificity indicate the tests’ ability to correctly distinguish between sick and healthy people.^(13)^

## DISCUSSION

### Detection of miRNA in human fluids

The detection of miRNAs in human fluids is a promising diagnostic and prognostic tool because of their tissue- and tumor-specific expression. Increased or decreased expression of such miRNAs is related to specific patterns in malignant or normal cells of the bladder and other organs.^(4)^ miRNAs can be packed and released through exosomes or extracellular vesicles, enhancing their stability in biofluids such as urine and plasma.^(11)^ Since these small cell membrane vesicles have a stable structure that protects them against enzymatic degradation, the miRNAs present inside them can be used to identify the characteristics of the tissue of origin.^(14)^

The urothelium is an extensive and highly specialized tissue that overlies the entire urinary tract. Therefore, it is in close contact with urine. Consequently, it is natural to consider using urine for genetic analysis of urothelial cells, with the additional advantage of being easy to collect. In addition, urine tends to have fewer diluted biomarkers than serum because of the greater volume and natural barriers of blood.^(15)^ Therefore, most studies on miRNA expression in BCa have used urine samples for analyses.

It is worth emphasizing the particular challenge of differentiating interstitial cystitis from BCa using cystoscopy, as both conditions produce similar macroscopic urothelial changes.^(16)^ Therefore, in such instances, genetic and epigenetic tools that use urine as the source of material can be used for differential diagnosis. For example, miRNAs, such as miR-373-5p, miR-6766-5p, or even urinary protein expression are suitable.^(17,18)^

However, some authors have reported that blood as the source performs better as blood would have fewer pre-analytical and analytical problems than urinary samples. The miRNAs in plasma and serum samples have high stability, and cell-free plasma can be prepared using a simple two-step centrifugation.^(19)^ Chen et al.^(20)^ observed a significant association between the expression of a single miRNA (miR-101) in the serum of patients and the stage of the tumor, pathological grade, and lymph node metastasis, but not with age, sex, tumor size, or tumor stage.

### Multi-miRNA panels

By proposing that a single miRNA could be used to distinguish patients with BCa from healthy people, Wang et al.^(2)^ defend that a combination of several miRNAs might provide higher accuracy than individual miRNAs. Accordingly, they constructed a diagnostic panel combining the expression data of miR-130a-3p, miR-130b-3p, and miR-301a-3p (AUC, 0.961; sensitivity, 87.8%; specificity, 93.3%). Furthermore, they verified that the levels of miR-130a-3p and miR-301a-3p were significantly correlated with the tumor stage, while those of miR-130b-3p and miR-301b-5p were not.

Geva et al.^(21)^ also proposed a diagnostic score by summing the standardized levels of overexpressed miRNAs in urine. The expression of this panel was considerably higher in patients with urinary tract cancer than in healthy controls, with good discrimination ability (AUC: 0.89). A diagnostic panel, constructed based on a logistic regression model and comprising miR-132-3p, miR-7-5p, and miR-148b-3p extracted from blood, showed the potential to improve the accuracy of BCa diagnosis (AUC: 0.92; sensitivity, 90.24%; specificity, 81.71%). Additionally, miR-132-3p and miR-148b-3p were found to be associated with the prognosis of patients with BCa.^(22)^

Juracek et al.^(23)^ identified 76 miRNAs that were differentially expressed in the urine of patients with BCa and healthy individuals. The authors correlated the two most elevated urinary miRNAs to derive a score. This correlation enabled the distinction between patients and controls (sensitivity, 82%; specificity, 70%; AUC, 0.84), less advanced tumors (without invasion of the muscular layer), more advanced tumors (with invasion of the muscular layer), and the grade of tumor differentiation (low or high).

In the context of patients who present with hematuria, a common symptom in the diagnosis of bladder tumors, Piao et al.^(24)^ proposed the application of miRNAs to differentiate benign hematuria from that related to cancer. In their multicenter trial, Piao et al. evaluated urinary samples from patients with BCa and compared them to samples from patients with non-oncologic conditions (prostate benign hyperplasia, stress urinary incontinence, urolithiasis, and urinary tract infection). Based on the ratio of urinary miR-6124 and miR-4511, 284 patients with BCa were distinguished from 206 patients with hematuria or pyuria (sensitivity, 78.5%; specificity, 70.9%; AUC, 0.81). In patients with gross hematuria, the sensitivity improved to 94%. Hofbauer et al.^(25)^ also analyzed the expression of miRNAs in the urine of patients with BCa, using patients with microscopic hematuria as the control group, and composed a panel of six urinary miRNAs capable of distinguishing patients with BCa from those with microscopic hematuria (AUC: 0.88).

In a study conducted by Usuba et al.,^(26)^ seven candidate miRNAs were identified for BCa screening. The combination of these miRNAs demonstrated a high diagnostic precision for BCa (AUC, 0.97; sensitivity, 95%; specificity, 87%). Moreover, among the studies analyzed, this is the only study to investigate the performance of miRNAs in differentiating BCa from other types of cancers. For this, they analyzed miR-6087 alone and 2 miRNAs (miR-6087 and miR-1343-5p) and proposed a panel of 7 miRNAs that demonstrated high sensitivity (miR-6087, 94.4%; 2-miRNA, 94.9%; 7-miRNA, 95.4%) and specificity (84.5%) in discriminating BCa from twelve other solid tumors. Although there was no clear distinction between gastric adenocarcinoma and biliary tract cancer, the 7-miRNA panel demonstrated 100% specificity in differentiating between sarcoma, pancreatic cancer, and breast cancer.

### The use of miRNA as a diagnostic tool in patients with chronic kidney disease

The diagnosis of BCa in patients with chronic kidney disease is particularly challenging, therefore, often resulting in the disease advancing to a late stage. As some patients are anuric, VUC is not viable. Most of them cannot undergo contrast examinations such as urotomography.^(27)^ Due to these particular difficulties, some studies have focused on the expression of miRNAs in these patients.

Chen et al.^(28)^ specifically evaluated the serum expression of miRNAs for diagnosing BCa in patients with chronic kidney disease. They found that miR-19b-1-5b showed prognostic value, with patients having low levels of miR-19b-1-5b experiencing significantly shorter overall survival than those with high expression levels. The authors observed that five expression ratios of two miRNAs (miR-155-5p/miR-150-5p, miR-378a-3p/miR-150-5p, miR-636/miR-150-5p, miR-150-5p/miR-210-3p, and miR-19b-1/miR-378a-3p) were significantly different between cancer patients and controls. Subsequently, they combined four of these ratios in a multi-miRNA panel and obtained an AUC of 0.882.

Li et al.^(11)^ investigated the expression levels of miRNAs in the urine and plasma of patients with chronic kidney disease in Taiwan and established a relationship between miRNA expression and poor prognosis in urinary cancer. The authors also proposed a nomogram to validate the incidence of urothelial cancer (AUC, 0.73).

### Relationship between expression of miRNA and surgical ablation of tumors

Tölle et al.^(19)^ were the first to obtain plasma samples from the same patient at different disease stages for surveillance. In this study, when miR-15b-5p and miR-590-5p were combined based on a logistic regression model, no further improvement of the discrimination ability was achieved for them individually. A change in miRNA levels was not observed a few days after surgery for tumor ablation, which suggested them as potential biomarkers.

However, Yang et al.^(29)^ reported contradictory results. Plasma samples were collected from patients before and one month after surgery for bladder tumor resection to analyze the regulation of miR-10a-5p and compared to that of the healthy group (AUC, 0.81; sensitivity, 79.5%; specificity, 65.6%). The results showed that the plasma levels of miR-10a-5p were significantly lower one month after surgery than the initial values. A similar methodology was employed in a study performed by El-Shal et al.;^(30)^ urine samples, collected four weeks after surgery, confirmed a significant reduction in the expression of miR-96-5p and miR-183-5p.

### The use of miRNA in association with other diagnostic methods

There is no doubt that non-muscle-invasive BCa detection methods are not very specific, and thus, the use of new tools may represent an additional resource for diagnosing BCa in the earlier stages of the disease.^(31)^

Erdmann et al.^(4)^ compared the accuracy of nine miRNAs with that of VUC, a classic method for diagnosing BCa. None of these miRNAs surpassed the performance of a single VUC (sensitivity, 76.9%; specificity, 100%; accuracy, 84.0%). When VUC and four miRNAs (miR-125b, −145, −183, and −221) were used in association, there was an increase in diagnostic power (sensitivity, 84.6%; specificity, 95.7%; accuracy, 88%), suggesting a promising potential for the reliable diagnosis of BCa using urine samples. However, most patients with BCa in this study had high-grade tumors (83.7%), which may have increased the accuracy of the VUC.

The expression of urinary miRNA was also compared to VUC in a multicenter study by Piao et al.^(24)^ They found that the VUC sensitivity was only 7.8% for low-grade tumors, reaching up to 25% in patients with high-grade neoplasia. The sensitivity of the urinary biomarker studied was significantly higher than that of VUC, and they were able to detect BCa of any grade with a sensitivity of >70%.

Another successful association of miRNAs with traditional diagnostic methods in diagnosing BCa was reported by Jiang et al.^(32)^ They showed that miR-192 expression was significantly reduced in the urine of patients with BCa. This alteration was related to the stage and size of the tumor; the more advanced and larger the tumor, the lesser the miRNA expression. There was a significant increase in the sensitivity (93.2%) and specificity (76.7%) of this biomarker when combined with ultrasound. The combination of these two noninvasive methods showed no significant difference in sensitivity when compared to cystoscopy, an invasive examination, the gold standard for diagnosing BCa. Therefore, it may represent a promising substitute after validation in clinical studies.

### Limitations of the study

According to the hierarchical pyramid of evidence classification, the systematic review provided highly relevant evidence. Although only one database was searched, thorough research was conducted, with studies subjected to quality assessment following the criteria of a widely recognized assessment checklist. However, the usefulness of our study may be partially limited by the lack of a meta-analysis of the presented data. Nevertheless, the results presented here are relevant for future research.

## CONCLUSION

Recent evidence has shown that miRNAs are promising bladder cancer biomarkers that facilitate non-invasive diagnosis of bladder cancer. Diagnostic panels comprising multiple miRNAs have proven to be a viable strategy for accurate disease detection. The combination of miRNAs with other noninvasive diagnostic methods can improve their accuracy, thereby reducing the use of invasive methods and contributing to timely diagnosis.

## Data Availability

The datasets used and/or analyzed in the current study are available in this published article and its supplementary information files.
